# Relationship between anemia and occupational fall injuries in female part-time employees: an observational study of large supermarket stores in Japan

**DOI:** 10.1093/joccuh/uiae063

**Published:** 2024-10-29

**Authors:** Azusa Shima, Yuichiro Kawatsu, Ayumi Morino, Makoto Okawara, Keiki Hirashima, Naomi Miyamatsu, Yoshihisa Fujino

**Affiliations:** Department of Clinical Nursing, Shiga University of Medical Science, Seta-Tsukinowa-cho, Otsu 520-2192, Shiga, Japan; Occupational Health Care Office, HEIWADO Co Ltd, Nishiima-cho 1, Hikone 522-8511, Shiga, Japan; Department of Environmental Epidemiology, Institute of Industrial Ecological Sciences, University of Occupational and Environmental Health, Japan, 1-1 Iseigaoka Yahatanishi-ku, Kitakyushu 807-8555, Fukuoka, Japan; Occupational Health Care Office, HEIWADO Co Ltd, Nishiima-cho 1, Hikone 522-8511, Shiga, Japan; Department of Environmental Epidemiology, Institute of Industrial Ecological Sciences, University of Occupational and Environmental Health, Japan, 1-1 Iseigaoka Yahatanishi-ku, Kitakyushu 807-8555, Fukuoka, Japan; Occupational Health Care Office, HEIWADO Co Ltd, Nishiima-cho 1, Hikone 522-8511, Shiga, Japan; Department of Environmental Epidemiology, Institute of Industrial Ecological Sciences, University of Occupational and Environmental Health, Japan, 1-1 Iseigaoka Yahatanishi-ku, Kitakyushu 807-8555, Fukuoka, Japan; Department of Environmental Epidemiology, Institute of Industrial Ecological Sciences, University of Occupational and Environmental Health, Japan, 1-1 Iseigaoka Yahatanishi-ku, Kitakyushu 807-8555, Fukuoka, Japan; Department of Clinical Nursing, Shiga University of Medical Science, Seta-Tsukinowa-cho, Otsu 520-2192, Shiga, Japan; Department of Environmental Epidemiology, Institute of Industrial Ecological Sciences, University of Occupational and Environmental Health, Japan, 1-1 Iseigaoka Yahatanishi-ku, Kitakyushu 807-8555, Fukuoka, Japan

**Keywords:** occupational fall injuries, anemia, females

## Abstract

Objectives: Occupational fall injuries have recently increased markedly in Japan, together with an increase in later-middle-aged females in the labor market. However, the association between anemia, which is prevalent among Japanese females, and falls is unclear. Here, we investigated the association between anemia and occupational fall injuries.

Methods: Participants were 6780 part-time female employees aged 35-64 working in Japanese supermarket stores of a retail company and who had at least 1 health checkup each year between 2017 and 2022. Anemia was defined as hemoglobin (Hb) <12.0 g/dL (mild: Hb = 11.0-11.9 g/dL, moderate-severe: Hb < 11.0 g/dL). Fall injuries were defined as slips, trips, and falls on the same level, requiring medical attention based on the occupational injury data by the company. Incidence rate ratios (IRRs) for falls were estimated using multilevel Poisson regression, adjusting for age and body mass index.

Results: The annual rate of occupational fall injuries was 0.7%. The adjusted IRR for occupational fall injuries among participants with anemia was 1.71 (95% CI, 1.12-2.60). When dividing anemia into 2 groups, IRR was 1.46 (95% CI, 0.84-2.53) for mild anemia and 2.13 (95% CI, 1.18-3.84) for moderate-severe anemia (*P* for trend = .007).

Conclusions: In this observational study of employees of large Japanese supermarket stores, anemia was significantly associated with a higher incidence of occupational falls. Our findings suggest the importance of anemia in the prevention of occupational falls.

## Teaser

Key points

What is already known on this topic: Anemia is one of the risk factors for falls in the older population. However, this topic has not been investigated in workplaces, where the incidence of occupational fall injuries has increased significantly in recent years, especially among female employees.

What this study adds: A considerable association between anemia and occupational fall injuries on the same level among part-time female employees of supermarket stores was indicated for the first time. We also found a trend indicating that the risk of fall injuries was higher in those with severe anemia.

How this study might affect research, practice, or policy: The results suggest the importance of anemia in occupational safety. The prevalence of anemia in Japanese women is higher than in Western countries, and anemia is often neglected because of the lack of clear symptoms, even though it is usually correctable. The number of female workers in the workforce will increase substantially in the future. Fall prevention has been mainly implemented by safety professionals in terms of workplace environment, safety education, and physical fitness. Further studies are warranted on occupational safety for women from the perspective of their physical characteristics and specific health issues.

## 1. Introduction

Occupational falls are an important health concern. In 2017, the number of fatal occupational falls worldwide was estimated at 36 000.^1^ Even nonfatal falls can cause serious injuries, such as disability or fracture, as well as injuries requiring days away from work. Indeed, falls account for nearly 20% of nonfatal occupational injuries resulting in days away from work in many countries.^2^^,^^3^ Occupational falls involve workers’ compensation and medical costs, which in the United States have been estimated to incur annual costs of $70 billion,^4^ and in 37% of cases to require 31 or more days away from work.^2^ Furthermore, falls at working age may affect the family and community roles of patients.

Occupational fall injuries occurring on the same level (ie, on a horizontal surface) are a serious issue, with a worldwide increase especially among middle-aged and older female workers.^5^^‑^^7^ In Japan, female workers aged 50 and older, who are at high risk of falling, account for 18% of all workers.^8^ Falls on the same level are the most common occupational injury requiring days away from work (27%), and incidence is increasing due to the increasing number of female workers and the aging of the workforce.^9^ To date, fall prevention in Japanese workplaces has been conducted with great enthusiasm, mainly focused on the workplace environment, safety behaviors, and exercises to improve physical performance.^10^^‑^^12^ However, considering the current prevalence of falls in middle-aged and older female workers, greater attention should be focused on the physical condition of these workers.

Previous studies have identified several physical conditions associated with occupational falls, including obesity; sensory, motor, and cognitive dysfunction; and frailty.^6^^,^^13^^,^^14^ However, the association between anemia and occupational falls in the working-age population is unclear, notwithstanding several reports of a suggested association in older adults.^15^^‑^^19^ In Japan, the prevalence of anemia among females was reported as 17.3% in a study in urban areas,^20^ and as 14% in the 2019 National Health and Nutrition Survey,^21^ both higher than in other developed countries.^22^ It appears plausible that fatigue and reduced attention due to anemia are related to falls.

Here, we aimed to investigate the association between anemia and occupational fall injuries on the same level in female employees in large Japanese supermarket stores. Our hypothesis was that employees who had anemia in a health checkup would have more fall injuries in the following year.

## 2. Methods

### 2.1. Study setting and participants

The study was conducted at a retail company with 155 supermarket stores located across 9 prefectures in western Japan. About 90% of the employees of this company work in stores, and 60% of the store employees are female part-time; of these, 90% are aged 35-64. In accordance with the Industrial Safety and Health Act,^23^ the company provides annual health checkups for employees who work for more than 6 hours, with a 100% take-up rate (long-term absences due to illness or other reasons are exempted). Health checkups included anthropometry measurements, blood pressure measurements, blood tests (ages 35 years and older), a urine dipstick test, electrocardiogram, chest X-ray, and self-administered questionnaires on health-related habits, medical history, and presence or absence of menstruation.

The present study included part-time female employees aged 35-64 years old working at one of the supermarket stores and who had had at least 1 health checkup (conducted annually from January to March) during 2017-2022. We included those aged 35 years or older because they routinely had received blood tests in their health checkups. Participants were excluded if they had retired by April 1, were pregnant, or if they had missing information on anemia and menopausal status. The reason for including part-time employees only was that they work mainly in the standing position on the sales floor or in the stock room, and carrying loads was considered to carry a high risk of falls.^6^ Their jobs included the following: keeping stock; stocking grocery, daily necessity, and clothing shelves; packing fresh foods such as meat, fish, and vegetables; preparing sushi and boxed lunches; serving customers; and working around the cash register.

### 2.2. Data sources and ethics

Anemia was detected using a blood test from the annual health checkup conducted in the 155 supermarket stores and defined as a hemoglobin (Hb) level less than 12.0 g/dL, in accordance with the definition of the World Health Organization (WHO).^22^^,^^24^ For detailed analysis, participants were divided into 2 anemia groups: mild (Hb: 11.0-11.9 g/dL) and moderate-severe (Hb < 11.0 g/dL).^22^^,^^24^

The primary outcome was fall injuries occurring in the workplace. Fall injuries were defined as an occupational slip, trip, and fall in which contact with the source of injury occurred at approximately the same or a higher level than the surface supporting the injured person, using accident type classification tables for occupational injuries in Japan.^25^ In Japan, injuries requiring a visit to a hospital/clinic and which occurred at the workplace or on the way to work are covered by occupational accident insurance, based on the Industrial Accident Compensation Insurance Act.^26^ The number of occupational falls was obtained from data on occupational injuries held by the retail company.

In the department of occupational safety and health of the company, health checkup data (January-March) and the number of fall injuries occurring during the year after the checkup (April to March of the following year) were merged for each individual, anonymized, and used for occupational accident prevention. Six years of stored data sets were provided to us for the present study. Data on the severity of injury and the number of days absent from work were not included.

We adhered to the Strengthening the Reporting of Observational Studies in Epidemiology (STROBE) guideline for cohort studies.^27^ The study was conducted with the approval of the ethics committee of Medical Research, University of Occupational and Environmental Health, Japan (reference no. ER23-001).

### 2.3. Statistical analysis

First, we described the characteristics of the participants by anemia status. The presence or absence of menstruation was indicated because it was considered to be closely related to anemia. In the main analysis, we estimated incidence rate ratios (IRRs) and 95% CIs using multilevel Poisson regression, accounting for individual overlap. In the main analysis, the independent variable was anemia, and the dependent variable was the number of falls. The adjusted variables were age and body mass index (BMI), following previous findings that falls were more common in people with obesity.^6^ In addition, to investigate trends associated with the severity of anemia, we conducted analyses in which anemia was divided into 2 levels of severity.^24^

All data were analyzed using Stata 17 (StataCorp LLC, College Station, TX, USA). All reported *P* values are 2-tailed, and *P* < .05 was considered to indicate statistical significance.

## 3. Results

The number of female employees of the supermarket stores who had at least 1 health checkup in 2017-2022 was 12 641. Of these, 6793 were part-time employees aged 35-64 years. We observed 6780 (person years: 27 208) without missing value. Table 1 summarizes participant characteristics by the presence or absence of anemia. The mean (SD) age of the participants was 51.1 (8.1) years. Participants with anemia accounted for 11.7%. Of the 3198 premenopausal participants, anemia was observed in 644 (20.1%).

**Table 1:** Participant characteristics by anemia status, based on first-year data for those observed for more than 1 year.

	**Total** **(*n* = 6780)**	**No anemia** **(*n* = 5985)**	**Anemia** ^a^
**Age, mean (SD), y**	51.1 (8.1)	51.6 (8.1)	47.0 (7.0)
**Body mass index (SD), kg/m** ^**2**^	22.9 (4.4)	22.9 (4.4)	22.6 (4.4)
**Premenopausal, *n* (%)**	3198 (47.2)	2554 (42.7)	644 (81.0)
**Smoking status, *n* (%)**			
**Never**	4561 (67.3)	4008 (67.0)	553 (69.6%)
**Quit**	904 (13.3)	809 (13.5)	95 (11.9%)
**Current**	1315 (19.4)	1168 (19.5)	147 (18.5%)
**Daily drinker, *n* (%)**	599 (8.8)	550 (9.2)	49 (6.2)

a Anemia was defined as a hemoglobin level less than 12.0 g/dL.

There were 199 fall injuries in total, of which only 1 person fell twice. Table 2 shows the prevalence of occupational fall injuries with and without anemia and the IRR (95% CI) of falls with anemia compared with that of falls without anemia. After adjustment for age and BMI, the IRR for occupational falls among participants with anemia was 1.71 (95% CI, 1.12-2.60).

**Table 2:** IRR (95% CI) for occupational falls among female employees aged 35-64 years with and without anemia.

			**Age-adjusted**			**Age- and BMI-adjusted**		
	**Person years**	**Incidence (%)**	**IRR**	**95% CI**	***P***	**IRR**	**95% CI**	***P***
No anemia	24 285	172 (0.71)	reference			reference		
Anemia	2923	27 (0.92)	1.67	1.10-2.54	.017	1.71	1.12-2.60	.013

Abbreviations: BMI, body mass index; IRR, incidence rate ratio.

Table 3 shows the results after anemia was divided into 2 levels of severity. The IRR was 1.46 (95% CI, 0.84-2.53) for the group with mild anemia and 2.13 (95% CI, 1.18-3.84) for the group with moderate-severe anemia (*P* for trend = .007).

**Table 3:** IRR (95% CI) for occupational falls among female employees aged 35-64 years, according to anemia status.^a^

			**Age-adjusted**			**Age- and BMI-adjusted**		
	**Person years**	**Incidence (%)**	**IRR**	**95% CI**	***P*** ^**b**^	**IRR**	**95% CI**	***P*** **^b^**
								
**Without anemia**	24285	172 (0.71)	reference			reference		
**Mild anemia**	1636	14 (0.86)	1.42	0.82-2.47		1.46	0.84-2.53	
**Moderate-severe anemia**	1287	13 (1.01)	2.09	1.16-3.76		2.13	1.18-3.84	
					.010			.007

Abbreviations: BMI, body mass index; Hb, hemoglobin; IRR incidence rate ratio.

a Anemia status: without anemia (Hb ≥ 12.0 g/dL), mild anemia (Hb: 11.0-11.9 g/dL), and moderate-severe anemia (Hb < 11.0 g/dL).

b *P* for trend.

## 4. Discussion

In this study, we found a considerable association between anemia and occupational falls on the same level among part-time female employees aged 35-64 working in Japanese supermarket stores. We also found a trend that the risk of fall injuries was higher among those with moderate-severe anemia. To our knowledge, this is the first study to report an association between anemia and occupational fall injuries in female employees. The results suggest the importance of anemia, an issue of physical health condition, as well as safety aspects, in the prevention of occupational falls.

Anemia is considered to increase the risk of falls via the following mechanisms. First, anemia is characterized as an abnormally low Hb concentration, and the resulting tissue hypoxia induces symptoms directly related to falls, such as fatigue, weakness, dizziness, and loss of physical working capacity.^28^^,^^29^ Second, anemia is reported to induce a decrease in cognitive ability and poor concentration, particularly in iron deficiency anemia.^30^^,^^31^ Reduced attention to one’s feet would facilitate slipping and tripping. Third, anemia can be a proxy for serious health conditions and underlying illness. Physical conditions reported to cause anemia include nutritional imbalance, inflammatory bowel disease, hematologic diseases, and cancer, which degrade physical functioning. Workers with these conditions may be at risk of falls. The trend that severe anemia was associated with a high incidence of fall injuries also suggests that anemia increases falls risk through a worsening of symptoms and cognitive function. The reason that significantly more fall injuries were not observed in the mild anemia group was probably due to lack of statistical power. Further studies in larger populations are warranted.

The characteristics of the work tasks commonly undertaken by the participants in this study may have emphasized the association between anemia and fall injuries. Participants were engaged in multitask walking in supermarket stores, including carrying loads and providing customer service.^6^^,^^32^ Falls are caused by the complex interaction of multiple factors, including personal, environmental, and work-related factors.^33^ Participants might have been affected by tasks more likely to have induced falls. For example, the association between anemia and falls is less likely to be observed in office workers. In Japan, approximately 7.5 million people (11% of all workers) work in the retail industry,^34^ where falls account for 37% of all occupational injuries.^9^ Indeed, occupational falls in the retail industry are prevalent worldwide. Falls are also a serious problem for health care workers who work in standing positions, particularly in late-middle-aged females.^4^ Closer attention to the risk of anemia as a cause of occupational falls is required in workplaces where the falls hazard is substantial. Further studies are warranted on the association between anemia and falls in different workplaces and demographic groups.

Our present results raise the important issue of anemia in females in occupational health. The prevalence of anemia among premenopausal participants in the present study was 20.1%, which was similar to that in the general Japanese population and in nonpregnant Asian females aged 15-49 years,^20^^,^^21^ and higher than in Western countries.^22^ Moreover, 44% of workers with anemia had moderate-severe anemia. They might not have received treatment, despite anemia being in many cases treatable. This was probably because the symptoms of chronic anemia are often camouflaged by biological compensatory mechanisms. Recent attention has focused on the finding that many premenopausal females have iron deficiency, a pre-stage of iron deficiency anemia, and that iron deficiency itself is associated with a multitude of symptoms that affect both mental and physical health and work productivity.^29^^,^^35^ Further, other findings have suggested that treatment for heavy menstrual bleeding and iron deficiency improves fatigue, physical function, and cognitive function in females, in addition to blood variables.^31^^,^^36^^‑^^38^ Blood tests for iron deficiency are not stipulated in Japanese occupational health checkups, and our present participants were not screened for iron deficiency or heavy menstrual flow. Given that the employment rate among Japanese females is currently over 70% and still increasing, occupational health should be more closely concerned with health risks for females.^39^

Our study had several limitations. First, any generalization of the results should be done with caution, given that the study was conducted in the supermarket stores of a retail company. The company was relatively large and maintained a policy focus on the work environment and safety training. The risk of falls may have been lower than in companies with less concern about fall prevention. Conversely, compared with the present setting it is possible that the impact of anemia is greater in work environments with a high fall hazard. Second, we did not consider the type of tasks, their work environment, and shifts. For example, the fall hazard would differ for workers handling produce in wet environments compared with cashiers in the clothing section. These effects were uncertain. Nevertheless, we found only a few studies limited to specific tasks or work environments,^40^ perhaps owing to the infeasibility in sample size. It is even more difficult to investigate the effects of anemia in a limited setting. Third, any improvement or worsening of anemia consequent to a health checkup may have affected the results. However, given that we assessed the association between anemia and falls on an annual basis, this effect would not be large. In addition, such change in anemia status would attenuate the results, suggesting that the actual association may be stronger. Fourth, although the causes and physical impact of anemia may differ before and after menopause, the effects of menopause were not considered. Actually, in the participants with anemia, 50% of those before menopause had mean corpuscular volume (MCV) <80fL, suggesting iron deficiency anemia, whereas only 5% of those after menopause had MCV <80fL. However, we did not observe an interaction between menopause and anemia in our preliminary analysis. The effects of menopause were unknown. Fifth, we could not consider the impact of individual physical peculiarity such as imbalance, behavioral factors, and specific triggers for falls (slips, trips).

Allowing for these limitations, it should nevertheless be noted that anemia was associated with occupational fall injuries. It is not known whether an improvement in anemia will decrease falls. The association between anemia and falls should be investigated in more detail, from different standpoints and in different populations.

## 5. Conclusion

In this observational study in large Japanese supermarket stores, we investigated the association between anemia and occupational fall injuries in part-time employees aged 35-64 years. We observed that anemia was significantly associated with a higher incidence of occupational falls. Our finding suggests the importance of treating anemia to prevent occupational falls.

## Data Availability

The company in which this study was conducted did not agree for their data to be shared publicly, so supporting data are not available.

## References

[1] Stanaway JD , Afshin A, Gakidou E, et al. Global, regional, and national comparative risk assessment of 84 behavioural, environmental and occupational, and metabolic risks or clusters of risks for 195 countries and territories, 1990–2017: a systematic analysis for the Global Burden of Disease study 2017. Lancet. 2018;392(10159):1923-1994. 10.1016/S0140-6736(18)32225-6 30496105 PMC6227755

[2] U.S. Bureau of Labor Statistics . Injuries, illnesses, and fatalities. Accessed October 22, 2023. https://www.bls.gov/iif/

[3] European Agency for Safety and Health at Work . Accidents and incidents. Accessed October 23, 2023. https://oshwiki.osha.europa.eu/en/themes/accidents-and-incidents

[4] Centers for Disease Control and Prevention . Falls in the workplace. Workplace safety and health topics. Accessed October 1, 2023. https://www.cdc.gov/niosh/falls/about/index.html?CDC_AAref_Val=https://www.cdc.gov/niosh/topics/falls/default.html#:%7E:text=

[5] Yeoh HT , Lockhart TE, Wu X. Non-fatal occupational falls on the same level. Ergonomics. 2013;56(2):153-165. 10.1080/00140139.2012.746739 23216368 PMC3578063

[6] Chang WR , Leclercq S, Lockhart TE, Haslam R. State of science: occupational slips, trips and falls on the same level. Ergonomics. 2016;59(7):861-883. 10.1080/00140139.2016.1157214 26903401 PMC5078727

[7] Scott KA , Fisher GG, Barón AE, Tompa E, Stallones L, DiGuiseppi C. Same-level fall injuries in US workplaces by age group, gender, and industry. Am J Ind Med. 2018;61(2):111-119. 10.1002/ajim.22796 29193187

[8] Ministry of Internal Affairs and Communications . Table 3-3: Employed person and employment rate by age (five-year group). Labour Force Survey. Accessed August 7, 2022. https://www.e-stat.go.jp/en/stat-search/files?page=1 layout=datalist toukei=00200531 tstat=000000110001 cycle=0 tclass1=000001040276 tclass2=000001011681 tclass3val=0

[9] Ministry of Health, Labour and Welfare, Japan . Analysis of occupational accidents in 2022 [in Japanese]*.* Accessed July 13, 2023. https://www.mhlw.go.jp/stf/newpage_33256.html

[10] Ministry of Health, Labour and Walfare, Japan . STOP falls project! [in Japanese]. Accessed October 1, 2023. https://anzeninfo.mhlw.go.jp/information/tentou1501_20.html

[11] Ministry of Health, Labour and Welfare, Japan . The 14th occupational safety & health program. Accessed September 20, 2024. https://www.mhlw.go.jp/content/11200000/001253683.pdf

[12] Nakamura T , Oyama I, Fujino Y, et al. Evaluation and simplification of the occupational slip, trip and fall risk-assessment test. Ind Health. 2016;54(4):354-360. 10.2486/indhealth.2015-0125 27021057 PMC4963548

[13] Osuka Y , Okubo Y, Nofuji Y, et al. Modifiable intrinsic factors related to occupational falls in older workers. Geriatr Gerontol Int. 2022;22(4):338-343. 10.1111/ggi.14370 35266260

[14] Matsugaki R , Matsuzaki H, Saeki S, Fujino Y, Matsuda S. Frailty and occupational falls among older Japanese workers: an internet-based cross-sectional study. J Occup Health. 2023;65(1):e12424. 10.1002/1348-9585.12424 37715321 PMC10504425

[15] Dharmarajan TS , Norkus EP. Mild anemia and the risk of falls in older adults from nursing homes and the community. J Am Med Dir Assoc. 2004;5(6):395-400. 10.1016/S1525-8610(04)70008-0 15530178

[16] Penninx BWJH , Pluijm SMF, Lips P, et al. Late-life anemia is associated with increased risk of recurrent falls. J Am Geriatr Soc. 2005;53(12):2106-2111. 10.1111/j.1532-5415.2005.00491.x 16398894

[17] Dharmarajan TS , Avula S, Norkus EP. Anemia increases risk for falls in hospitalized older adults: an evaluation of falls in 362 hospitalized, ambulatory, long-term care, and community patients. J Am Med Dir Assoc. 2007;8(3 suppl 2):e9-e15 17352999 10.1016/j.jamda.2006.12.015

[18] Hopstock LA , Utne EB, Horsch A, Skjelbakken T. The association between anemia and falls in community-living women and men aged 65 years and older from the fifth Tromsø study 2001-02: a replication study. BMC Geriatr. 2017;17(1):292. 10.1186/s12877-017-0689-8 29282000 PMC5745627

[19] Kim Y , Kim J. Relationship between anemia and falls among postmenopausal women in Korea. Int J Environ Res Public Health. 2022;19(14). 10.3390/ijerph19148242 PMC9316311 35886093

[20] Kusumi E , Shoji M, Endou S, et al. Prevalence of anemia among healthy women in 2 metropolitan areas of Japan. Int J Hematol. 2006;84(3):217-219. 10.1532/IJH97.06097 17050194

[21] Ministry of Health, Labour and Welfare, Japan . National health and nutrition survey Japan, 2019. Accessed September 1, 2023. https://www.mhlw.go.jp/stf/seisakunitsuite/bunya/kenkou_iryou/kenkou/eiyou/r1-houkoku_00002.html

[22] Stevens GA , Paciorek CJ, Flores-Urrutia MC, et al. National, regional, and global estimates of anaemia by severity in women and children for 2000–19: a pooled analysis of population-representative data. Lancet Glob Health. 2022;10(5):e627-e639. 10.1016/S2214-109X(22)00084-5 35427520 PMC9023869

[23] Ministry of Health, Labor and Welfare, Japan . Industrial Safety and Health Act*.* Accessed September 11, 2023. https://www.japaneselawtranslation.go.jp/en/laws/view/3440

[24] World Health Organization . Haemoglobin concentrations for the diagnosis of anaemia and assessment of severity. Accessed November 13, 2023. https://apps.who.int/iris/bitstream/handle/10665/85839/WHO_NMH_NHD_MNM_11.1_eng.pdf

[25] Ministry of Health, Labour and Welfare . Accident type classification tables for occupational injuries [in Japanese]. Accessed October 15, 2023. https://jsite.mhlw.go.jp/yamagata-roudoukyoku/var/rev0/0114/4823/2015717113029.pdf

[26] Ministry of Justice, Japan . Compensation Insurance Act. Accessed October 22, 2023. https://www.japaneselawtranslation.go.jp/ja/laws/view/3879

[27] STROBE. Strengthening the reporting of observational studies in epidemiology (STROBE) . Accessed September 15, 2024. https://www.strobe-statement.org/

[28] Domenica Cappellini M , Motta I. Anemia in clinical practice—definition and classification: does hemoglobin change with aging? Semin Hematol. 2015;52(4):261-269. 10.1053/j.seminhematol.2015.07.006 26404438

[29] Lopez A , Cacoub P, Macdougall IC, Peyrin-Biroulet L. Iron deficiency anaemia. Lancet. 2016;387(10021):907-916. 10.1016/S0140-6736(15)60865-0 26314490

[30] Murray-Kolb LE , Beard JL. Iron treatment normalizes cognitive functioning in young women. Am J Clin Nutr. 2007;85(3):778-787. 10.1093/ajcn/85.3.778 17344500

[31] Dugan C , MacLean B, Cabolis K, et al. The misogyny of iron deficiency. Anaesthesia. 2021;76(suppl 4):56-62. 10.1111/anae.15432 33682094

[32] Kodithuwakku Arachchige SNK , Chander H, Knight AC, Burch RFV, Chen CC, Reneker JC. Dual tasking during trip recovery and obstacle clearance among young, healthy adults in human factors research. Int J Environ Res Public Health. 2021;18(19):10144. 10.3390/ijerph181910144 PMC8507707 34639448

[33] Hsiao H . Fall prevention research and practice: a total worker safety approach. Ind Health. 2014;52(5):381-392. 10.2486/indhealth.2014-0110 25345424 PMC4246530

[34] Statistics Bureau, Ministry of Internal Affairs and Communications. Activity Survey survey results [in Japanese] . 2021 Economic Census for Business Activity. Accessed November 14, 2023. https://www.stat.go.jp/data/e-census/2021/kekka/index.html

[35] Benson CS , Shah A, Stanworth SJ, et al. The effect of iron deficiency and anaemia on women’s health. Anaesthesia. 2021;76(suppl 4):84-95. 10.1111/anae.15405 33682105

[36] Houston BL , Hurrie D, Graham J, et al. Efficacy of iron supplementation on fatigue and physical capacity in non-anaemic iron-deficient adults: a systematic review of randomised controlled trials. BMJ Open. 2018;8(4) e019240. 10.1136/bmjopen-2017-019240 PMC5892776 29626044

[37] Favrat B , Balck K, Breymann C, et al. Evaluation of a single dose of ferric carboxymaltose in fatigued, iron-deficient women—PREFER a randomized, placebo-controlled study. PLoS One. 2014;9(4):e94217. 10.1371/journal.pone.0094217 24751822 PMC3994001

[38] Peuranpää P , Heliövaara-Peippo S, Fraser I, Paavonen J, Hurskainen R. Effects of anemia and iron deficiency on quality of life in women with heavy menstrual bleeding. Acta Obstet Gynecol Scand. 2014;93(7):654-660. 10.1111/aogs.12394 24912842

[39] Gender Equality Bureau Cabinet Office, Japan. Trends in the percentage of women in employment [in Japanese]. White Paper on Gender Equality 2023. Accessed November 14, 2023. https://www.gender.go.jp/about_danjo/whitepaper/r05/zentai/html/zuhyo/zuhyo02-01.html

[40] Verma SK , Lombardi DA, Chang WR, et al. Rushing, distraction, walking on contaminated floors and risk of slipping in limited-service restaurants: a case–crossover study. Occup Environ Med. 2011;68(8):575-581. 10.1136/oem.2010.056226 21097951

